# Woorara in Tetanus

**Published:** 1860-02

**Authors:** 


*Woorara in Tetanus.*—The action of this mysterious poison in relaxing the muscular system, has led to its trial in France and England in the treatment of tetanus. Some cases are reported as cured by this means, but a greater number in which it has been tried without success. The actual composition of this poison is not at all understood, and the pathology of the disease for which it is recommended is also involved in obscurity. This, of course, renders its use rather empirical, especially as no two specimens of woorara are alike in activity or in the nature of their effects upon the system. We should hardly recommend an extensive repetition of the experiments which have been made on this subject, especially as we have no means of counteracting the effects of the poison should they be too severe.

